# Meta-analysis of the efficacy and adverse drug reactions of adrenergic alpha-antagonists in treating children with ureteral calculi

**DOI:** 10.3389/fped.2023.1098002

**Published:** 2023-02-22

**Authors:** Kai Sun, Peizhi Zhang, Yanning Sun, Qingliang Wang, Qinghua Xia

**Affiliations:** ^1^ Department of Urology, Shandong Provincial Hospital, Shandong University, Jinan, China; ^2^ Department of Urology, Shandong Provincial Hospital Affiliated to Shandong First Medical University, Jinan, China

**Keywords:** adrenergic alpha-Antagonists, children, ureteral calculi, meta-analysis, randomized controlled trials

## Abstract

This meta-analysis investigated the efficacy and adverse drug reactions (ADRs) of three different adrenergic alpha-antagonists during the treatment of pediatric ureteral stones. Studies were retrieved from MEDLINE, EMBASE, and the Cochrane Controlled Trial Registry until January 2022. We identified 7 articles, including six RCTs and one cohort study. 610 children received either adrenergic alpha-antagonists or placebo. The results confirmed that the three different adrenergic alpha-antagonists could significantly increase the ureteral calculi expulsive rate and shorten the ureteral calculi expulsive time, regardless of the size of the stone “<5 mm” or “5–10 mm”. Subgroup analysis suggested that all three adrenergic alpha-antagonists increased the ureteral calculi expulsive rate. Tamsulosin and silodosin also have the effect of shortening ureteral calculi expulsive time, while doxazosin has an insignificant effect on ureteral calculi expulsive time. Besides, tamsulosin and silodosin obviously reduced the number of pain episodes caused by ureteral calculi in children. We analyzed the treatment-emergent adverse events (TEAEs) caused by the treatment of three different adrenergic alpha-antagonists to explore their ADRs. The probability of ADRs was increased after treatment with adrenergic alpha-antagonists. Further subgroup analysis revealed the application of tamsulosin was positively correlated with ADRs in children with ureteral calculi, while the application of doxazosin and silodosin had no statistically significant effect on the probability of TEAEs. In a conclusion, this article systematically analyzed the efficacy and ADRs of three different adrenergic alpha-antagonists, and provided reference and guidance for the application of adrenergic alpha-antagonists to treat children ureteral calculi.

## Introduction

A disorder of the deposition of crystallites in the urinary tract is defined as urolithiasis (1). An epidemiological survey showed that incidence of urolithiasis in children worldwide increased fivefold in the past 20 years (2). Considering the complex factors in pediatric stone disease, such as the etiology, location, size and composition of the stones, the treatment of ureteral calculi includes medical expulsive therapy (MET), shock wave lithotripsy (SWL), minimally invasive surgery such as ureteroscopy (URS) or percutaneous nephrolithotomy (PNL), and even open surgery (3). Furthermore, the recurrence rate of ureteral stones is still close to 50% due to the complexity of stone formation factors (4). Moreover, the trauma caused by repeated surgical treatment may not only endanger the health of patients but also may lead to an increased recurrence. Therefore, we should adhere to the principle of non-invasive or minimally invasive treatment on the premise of ensuring the good effect of patients with ureteral calculi (especially children), which encourages us to pay more attention to MET therapy (5).

At present, the reports on remedy of adults' ureteral calculi are quite extensive, and the appropriate mini-invasive treatments like SWL or URS according to the location and size of calculi often produces better results (6). However, SWLs cannot adequately gather energy in smaller children, meanwhile repeated SWLs can also cause children to suffer from side effects of repeated general anesthesia (7). URS is routinely used due to its advantages of less trauma and less pain, especially when calculi size ≥10 mm (8). However, patients with small stones experience greater trauma when undergoing invasive surgery, which is one of the disadvantages of invasive surgery over other non-invasive treatments (9). Therefore, effective non-invasive therapies are being developed for the treatment of pediatric ureteral stones. Currently, the drugs for MET treatment are mainly adrenergic alpha-antagonists, calcium channel blockers, and phosphodiesterase inhibitors. The effects and range of application of adrenergic alpha-antagonists appear to be superior to other drugs (10). Tamsulosin, doxazosin, and silodosin are the most representative adrenergic alpha-antagonists. Current studies on the treatment of ureteral calculi with adrenergic alpha-antagonists have focused on adults, while studies on children with ureteral calculi are limited and the samples are scattered (11). Therefore, we conducted a review and meta-analysis of existing studies on adrenergic alpha-antagonists for the treatment of ureteral calculi in children, in order to better understand the efficacy and adverse reactions of adrenergic alpha-antagonists, which can provide valuable clues for the treatment of ureteral calculi in children.

In this meta-analysis, we concluded that adrenergic alpha-antagonists can significantly increase the rate of ureteral stone expulsion in children regardless of stone size. At the same time, tamsulosin and silodosin were more advantageous in shortening the ureteral calculi expulsive time and reducing the number of pain attacks. In terms of safety, tamsulosin increased the incidence of TEAEs, but all were manageable. These results may provide guidance for us to select adrenergic alpha-antagonists in the treatment of pediatric ureteral stones.

## Materials and methods

### Search strategy

We conducted literature retrieval under the guidance of the Preferred Reporting Items for Systematic Reviews and Meta-Analyses (PRISMA) (12). Two authors systematically searched until January 1, 2022 for all relevant studies on adrenergic alpha-antagonists as the remedy of children ureteral calculi. We screened and identified RCTs and cohort studies from MEDLINE, EMBASE, and the Cochrane Controlled Trials Register Medline based on multiple combinations of Medical Subject Heading (Mesh) terms. Search terms included “adrenergic alpha antagonists”, “ureteral stones”, “children”, “randomized controlled trials” and “cohort studies”. The review process was not limited by the language system. We used Endnote software to manage downloaded articles and removed duplicate articles. The third person arbitrates any disputes.

### Inclusion criteria and trial selection

**Figure 1:**
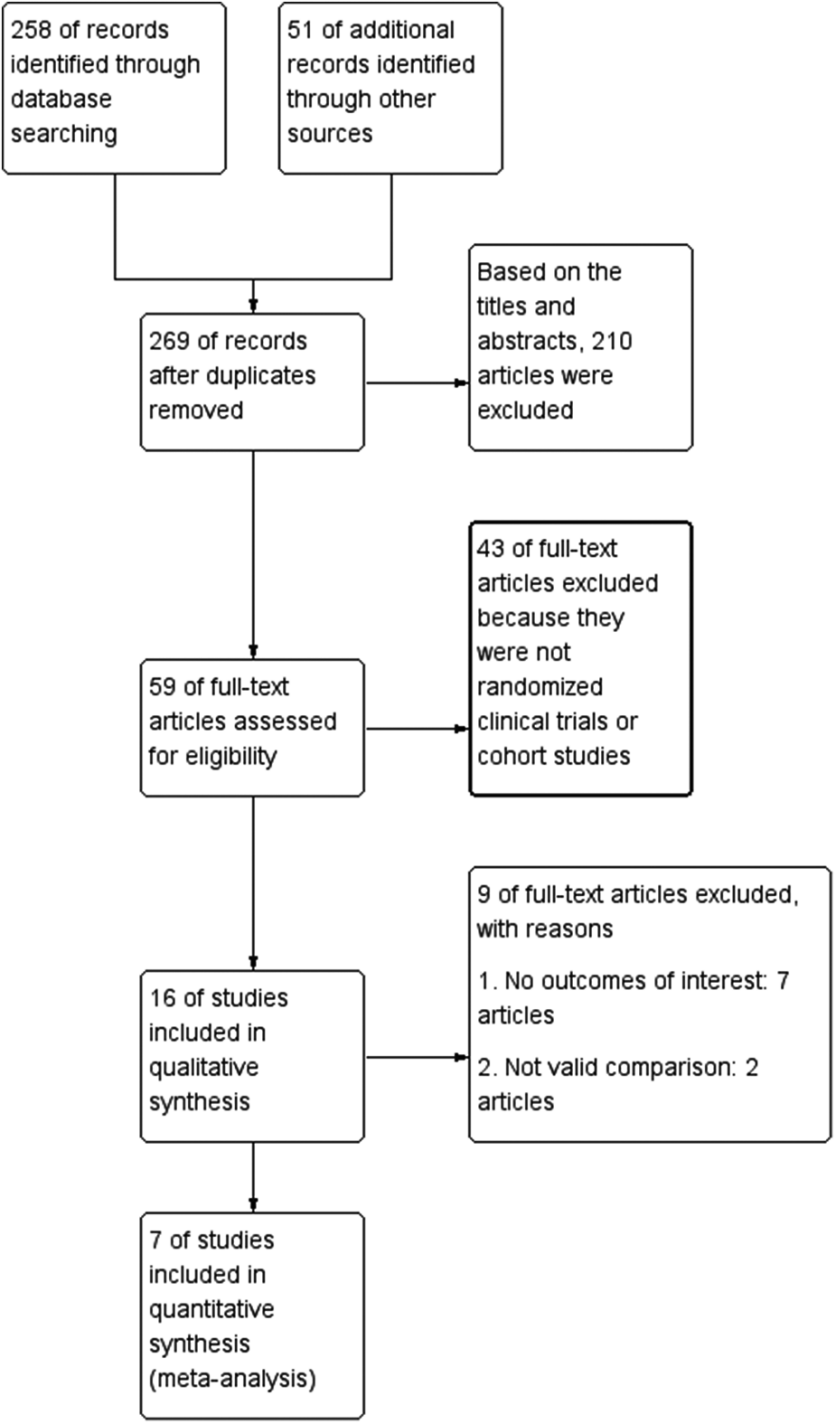
Flowchart of the study selection process.

The criteria for inclusion of studies were as follows: (a) Those associated with multiple adrenergic alpha-antagonists as remedy for children ureteral calculi; (b) Those 2–18 years as age inclusion indicators; (c) Those with full-text content; (d) Those with authentic data, chiefly incorporating the sum of subjects and each index relevant to the study content. Those involving the following factors were included once: (I) a group of subjects were studied by the same group of researchers; (II) the same group of subjects participated in extensive experiments. Others that did not meet the above criteria were excluded from this analysis. The selection flow chart was shown in Figure 1.

### Quality assessment

We used Cochrane bias risk evaluation facility compared with Jadad Score to assess the methodological quality of RCTs. The cohort study was evaluated based on the method of patient inclusion groups, exposure variables and covariates, sample size calculations, and propensity score matching. Each RCT or cohort study was classified based on quality assessment criteria: (1) This study included a low potential deviation from all quality standards; (2) This study was counted a secondary probability of meeting partial quality standards or fuzziness; (3) The study had a high probability of including bias from the bare quality criteria. The whole process by which all reviewers assess study quality was independent. Different opinions were discussed and an agreement was finally reached among reviewers.

### Data extraction

We first developed a data extraction tabulation to facilitate the systematic extraction of data included in articles by two independent authors. The extracted data included: (a) The name of first author; (b) Publication date; (c) Country of research; (d) The median age of the patients; (e) Types of drugs; (f) Sample size of patients in different group; (g) Administration method; (h) Treatment duration; (i) Dosage; (j) Main inclusion population; (k) Outcome measures. Our research did not require ethical consent.

### Statistical analysis

Review Manager version 5.3.0 (Cochrane Collaboration, Oxford, UK) was applied for statistics and data analysis for this study. Fixed or random effect models were suitable for judging indicators. Continuous outcomes and dichotomous outcomes were respectively compared by mean difference (MD) and Risk ratios (RR), along with 95% confidence interval (CI) for effect size. The effect of heterogeneity on the results was evaluated by I-square (*I*^2^) test. The application of fixed or random effect models was chosen based on the heterogeneity. If *I*^2^ value was greater than 50%, random effect model would be applied in the study. Provided *I*^2^ value was less than 50%, fixed effect model would be chosen. Subgroup analysis was utilized flexibly to analyze the sources of heterogeneity. *P* ≤ 0.05 was considered statistically significant.

## Results

### Study selection process, search results, and characteristics of trials

Reviewers first retrieved 269 unique articles from databases and other sources. After reviewing abstracts and titles and removing 210 articles that were irrelevant to our topic, the remaining 59 articles were downloaded in full and read in detail. Based on the rigor of experimental design and data reliability, we screened out 43 full-text articles because they were not RCTs or cohort studies. After screening out 7 articles that did not contain results of interest and 2 articles that did not include valid comparisons, two reviewers rated the filtered absolute papers separately and selected them according to the criteria. Finally, 6 RCTs and 1 cohort study were selected to explore the safety and effects of various adrenergic alpha-antagonists for remedy of children ureteral calculi. Table 1 described the contents and characteristics of the seven included studies (13–19).

**Table 1:** The details of included studies.

Study	Country	Age (median)	Therapy in experimental group	Therapy in control group	Sample size		Administration method	Treatment duration	Dosage	Main inclusion population	Quality assessment
					experimental group	control group					
Aydogdu et al. [2009]	Turkey	63.7	Doxazosin	Analgesics	19	20	Oral?	3 weeks	0.03 mg/Kg/d	Patients 2 to 14 years with DUS ≤10 mm	Low risk
Elgalaly et al. [2017]	Egypt	62.1	Silodosin	Analgesics	18	19	Oral	4 weeks	4 mg/d	Patients 5 to 17 years with DUS ≤10 mm	Low risk
Erturhan et al. [2012]	Turkey	63.8	Doxazosin	Analgesics	24	21	Oral	3 weeks	0.03 mg/Kg/d	Patients 3 to 15 years with DUS ≤10 mm	Low risk
Hussein A et al. [2015]	Egypt	66.0	Tamsulosin	analgesics	31	32	Oral	4 weeks	0.4 mg/d	Patients 2 to 15 years with DUS ≤10 mm	Low risk
Mohamed G et al. [2021]	Egypt	65.6	Silodosin/Tamsulosin	Analgesics	56/55	56	Oral	4 weeks	4 mg/d 0.4 mg/d	Patients 6 to 14 years with DUS ≤10 mm	Low risk
Mokhless et al. [2011]	Egypt	61.6	Tamsulosin	Analgesics	33	28	Oral	4 weeks	0.4 mg/d 0.2 mg/d	Patients 2 to 15 years with DUS ≤12 mm	Low risk
Tasian et al. [2014]	America	68.4	Tamsulosin	Analgesics	99	99	Oral	6 weeks	0.4 mg/d	Patients 2 to 18 years with DUS ≤10 mm	High risk

PDE5-Is, phosphodiesterase type 5 inhibitors; LUTS, lower urinary tract symptoms; BPH, benign prostatic hyperplasia; IPSS, international prostate symptom score; tPSA, prostate specific antigen; Qmax, maximum urine flow rate; QoL, quality of life; VAS, visual analogical scale; GAQ, global assessment quality; PVR, post-void residual; IIEF, international index of erectile function; MVV, minimum voided volume; ED, erectile dysfunction.

### Risk of bias in trials

**Figure 2:**
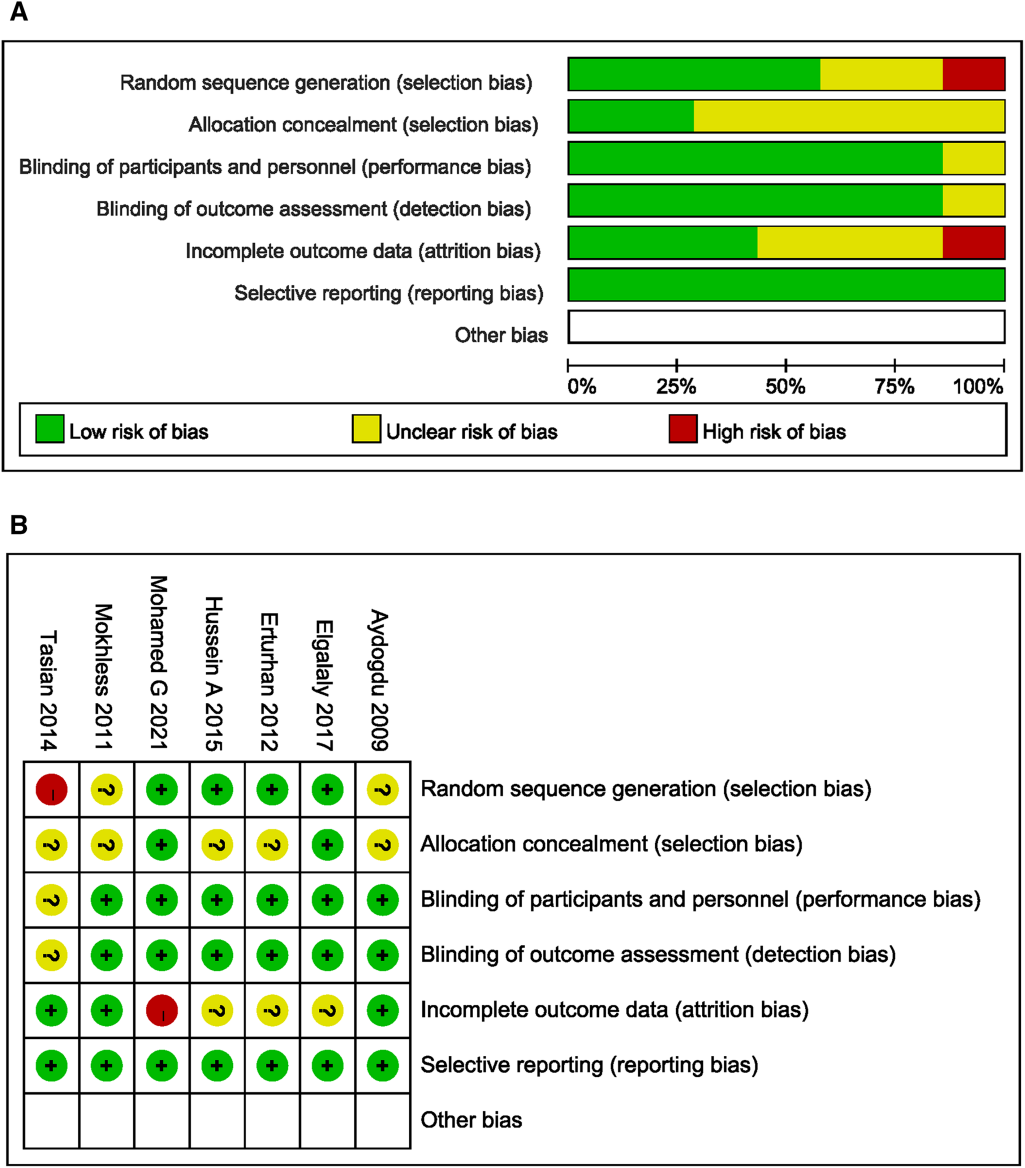
Bias risk assessment of included studies. (**A**) Risk of bias summary: Each bias risk item of all included studies was judged by review authors. (**B**) Risk of bias graph: The review authors quantified each bias risk item of included studies as a percentage.

The 7 included studies were 6 RCTs with a specific randomization protocol and 1 multi-institutional cohort study. We assessed the quality of them (Table 1). As differences in the doses of different adrenergic alpha-antagonists may have biased the results, we performed an analysis of the risk of bias, and produced a risk of bias map and summary (Figure 2).

### Ureteral calculi expulsive rate

**Figure 3:**
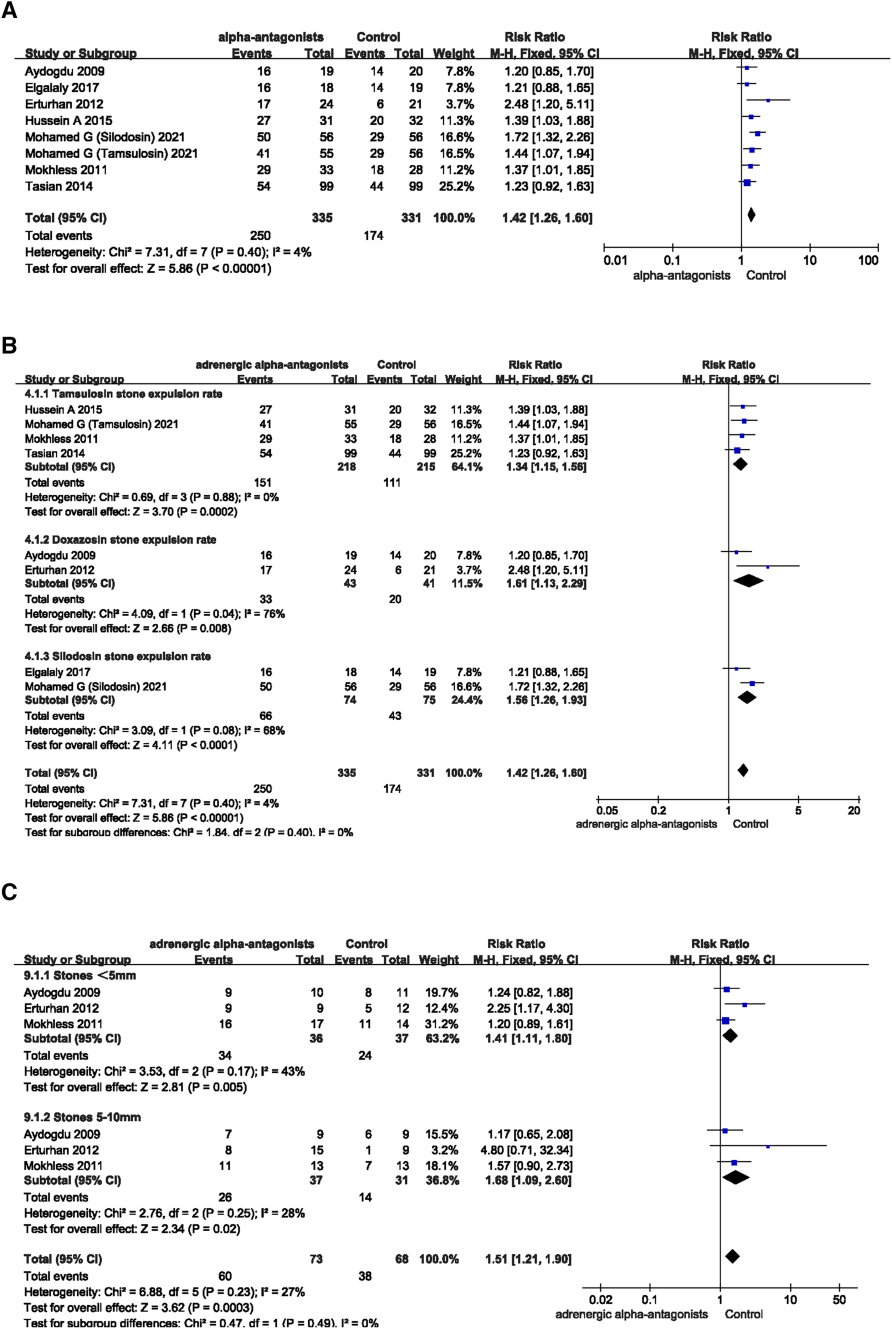
Forest plot comparing changes in ureteral calculi expulsive rate between adrenergic alpha-antagonists and control groups. (**A**) Effects of treatment with adrenergic alpha-antagonists on ureteral calculi expulsive rate compared with control group. (**B**) Subgroup analysis of the effects of adrenergic alpha-antagonists on the ureteral calculi expulsive rate. (**C**) Subgroup analysis of the effect of adrenergic alpha-antagonists on the expulsive rate of ureteral calculi of different sizes.

The ureteral calculi exclusion rate of children was counted in all 7 articles of the included studies. The forest plot showed the adrenergic alpha-antagonists group significantly increased the ureteral calculi expulsive rate, the pooled estimate of mean difference (MD) was 1.42, and the 95% confidence interval (CI) was (1.26 to 1.60) (*p* < 0.00001) (Figure 3A). Subsequent subgroup analyses by type of adrenergic alpha-antagonists in each study suggested tamsulosin (MD 1.34; 95% CI 1.15 to 1.56; *p* = 0.0002), doxazosin (MD 1.61; 95% CI 1.12 to 2.29; *p* = 0.008) and silodosin (MD 1.56; 95% CI 1.26 to 1.93; *p* < 0.0001) both increased the ureteral calculi expulsive rate in children (Figure 3B). Three of the included studies addressed the effect of stone diameter on the ureteral stone expulsive rate. According to the size of stone, children with ureteral calculi were divided into “<5 mm” “5–10 mm” two groups. The results of the forest plot suggested adrenergic alpha-antagonists could promote the rate of ureteral stone expulsion in children, regardless of the size of the stone “<5 mm” (MD 1.41; 95% CI 1.11 to 1.80; *p* = 0.005) or “5–10 mm” (MD 1.68; 95% CI 1.09 to 2.60; *p* = 0.02) (Figure 3C). These studies fully demonstrated the significant effects of various adrenergic alpha-antagonists on improving the ureteral calculi expulsive rate in children.

### Ureteral calculi expulsive time

**Figure 4:**
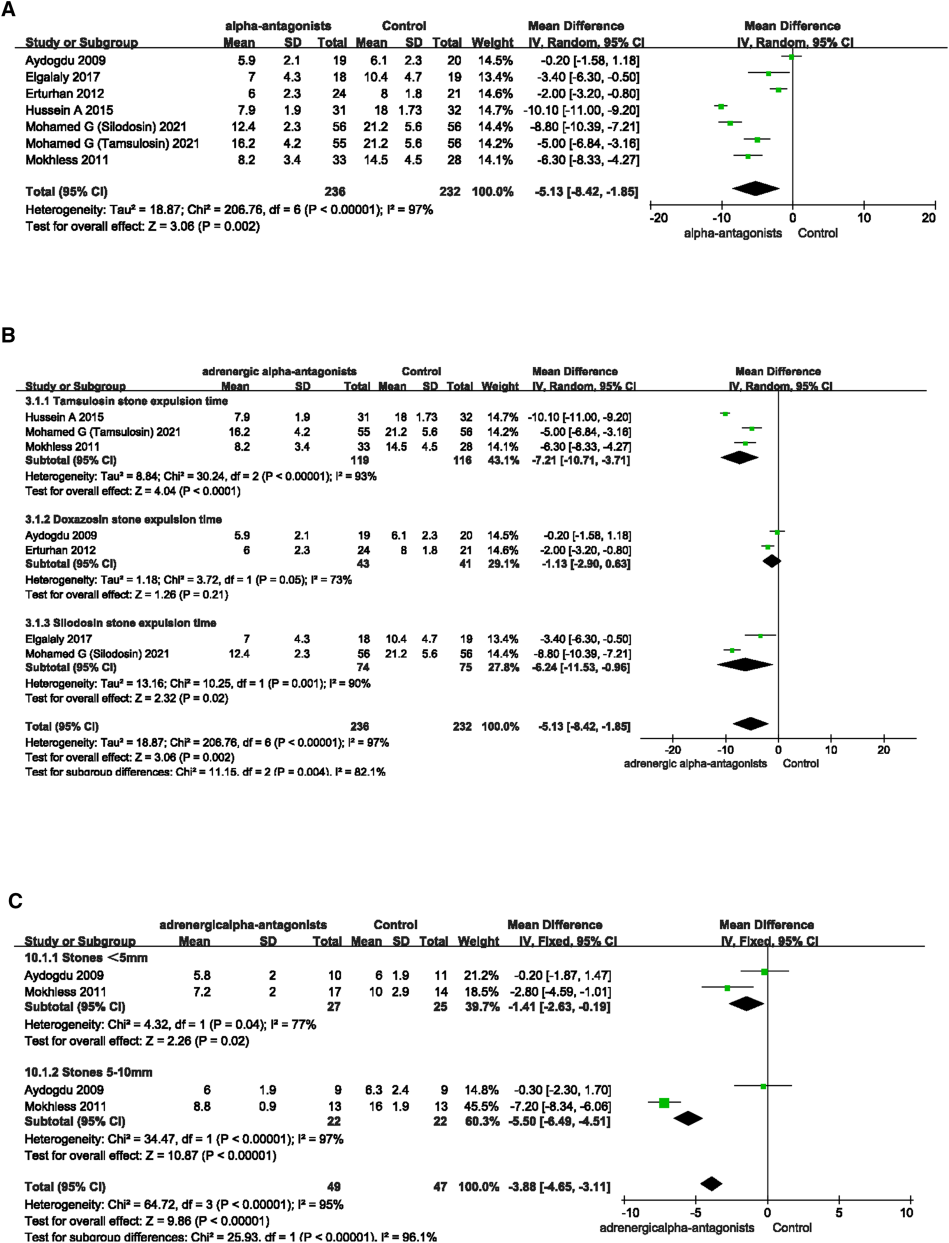
Forest plot comparing changes in ureteral calculi exclusion time between adrenergic alpha-antagonists and control groups. (**A**) Effects of treatment with adrenergic alpha-antagonists on ureteral calculi expulsive time compared with control group. (**B**) Subgroup analysis of effects of adrenergic alpha-antagonists on the ureteral calculi expulsive time. (**C**) Subgroup analysis of effects of adrenergic alpha-antagonists on the expulsive rate of ureteral calculi of different sizes.

We searched for and integrated data from the included articles for a total of 468 children to further investigate the effects of adrenergic alpha-antagonists on shortening ureteral calculi expulsive time in children. All patients included in this study included 236 patients in the adrenergic alpha-antagonists treatment group (119 patients in the tamsulosin, 43 patients in the doxazosin and 74 patients in the silodosin group) and 232 patients in the control group. The results showed that children treated with adrenergic alpha-antagonists had a significantly shorter time to ureteral stone expulsive than the control group (MD −5.13; 95% CI −8.42 to −1.85; *p* = 0.002), which was in line with our expectations (Figure 4A). Due to the heterogeneity of treatment effects among different adrenergic alpha-antagonists, subgroup analysis is performed to investigate the effects of tamsulosin, doxazosin, and silodosin on ureteral calculi expulsive time. Compared with the control group, the ureteral calculi expulsive time of children in the tamsulosin group (MD −7.21; 95% CI −10.71 to −3.71; *p* < 0.0001) and silodosin group (MD −6.24; 95% CI −11.53 to −0.96; *p* = 0.02) was significantly shortened (Figure 4B), while doxazosin treatment was insignificant (MD −1.13; 95% CI −2.90 to 0.63; *p* = 0.21). Two of the included studies investigated the effect of adrenergic alpha-antagonists on the ureteral calculi expulsive time of different sizes. We analyzed the relevant data in these two articles, and found the treatment of adrenergic alpha-antagonists could shorten the ureteral calculi expulsive time, regardless of the size of the stone “<5 mm” (MD −1.41; 95% CI −2.63 to −0.19; *p* = 0.02) or “5–10 mm” (MD −5.50; 95% CI −6.49 to −4.51; *p* < 0.00001) (Figure 4C).

### Number of pain episodes

**Figure 5:**
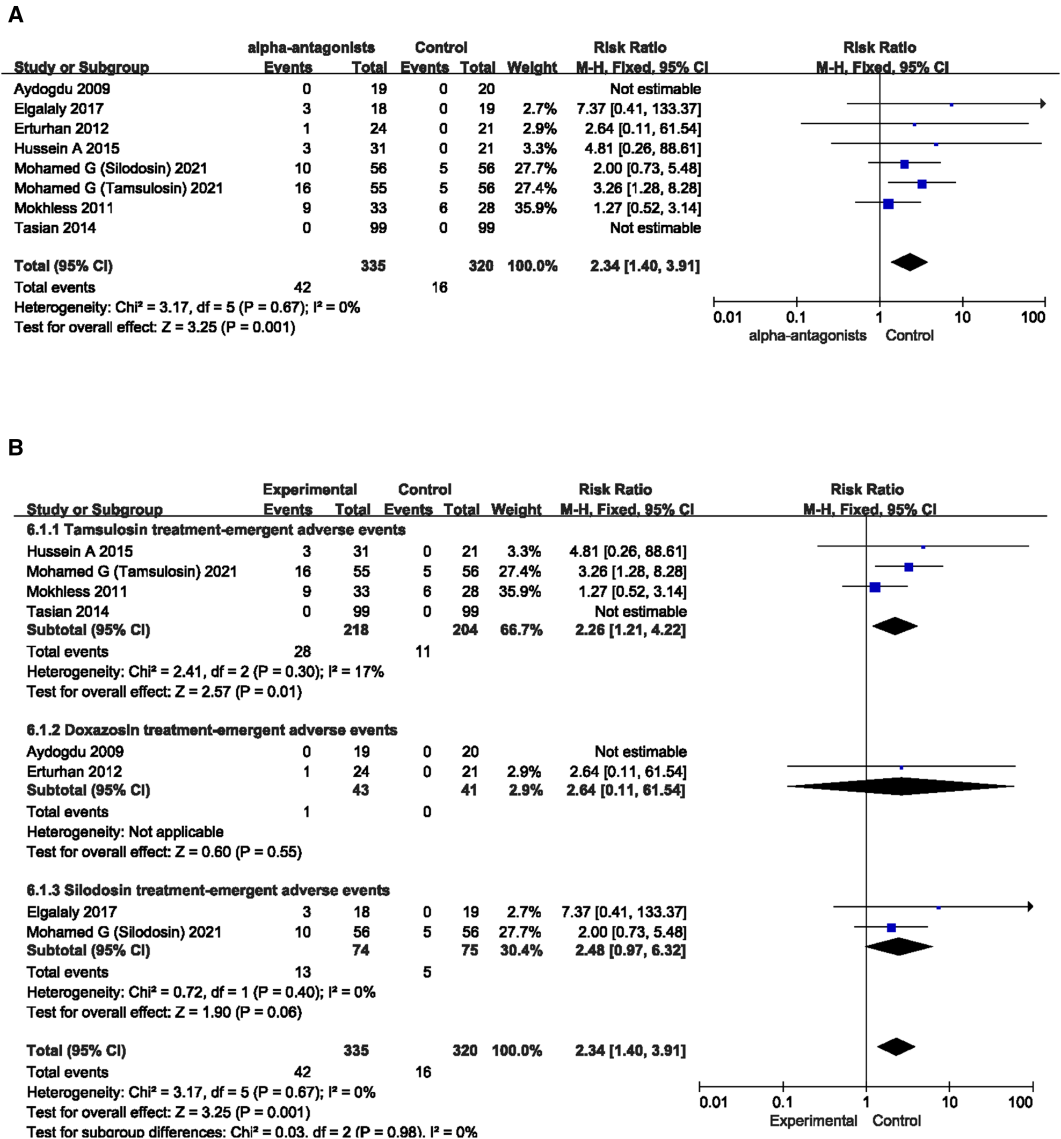
Forest plot comparing differences in treatment-emergent adverse events between 438 adrenergic alpha-antagonists and control groups. (**A**) Effects of treatment with adrenergic 439 alpha-antagonists on treatment-emergent adverse events compared with control group. (**B**) Subgroup analysis of the effects of adrenergic alpha-antagonists on the 440 treatment-emergent 441 adverse events.

To investigate the effect of adrenergic alpha-antagonists on pain relief from ureteral calculi, we screened 4 RCTs involving pain episodes from the included articles. The four RCTs involved two adrenergic alpha-antagonists, tamsulosin and silodosin. We first compared the effect of adrenergic alpha-antagonists on number of pain episodes with the control group. Forest plots suggested that adrenergic alpha-antagonists significantly reduced the frequency of pain episodes caused by ureteral stones in children, the pooled estimate of mean difference (MD) was −1.02, and the 95% confidence intervals (CI) was (−1.27 to −0.76) (*p* < 0.00001) (Figure 5A). In order to explore heterogeneity of different drugs in relieving pain episodes caused by ureteral stones, we further performed a subgroup analysis. The subgroup analysis results were as follows, tamsulosin (MD −0.87; 95% CI −1.21 to −0.54; *p* < 0.00001) and silodosin (MD −1.59; 95% CI −2.82 to −0.35; *p* < 0.0001), suggesting that both kinds of adrenergic alpha-antagonists can effectively relieve pain caused by ureteral stones.

### Treatment-emergent adverse events (TEAEs)

**Figure 6:**
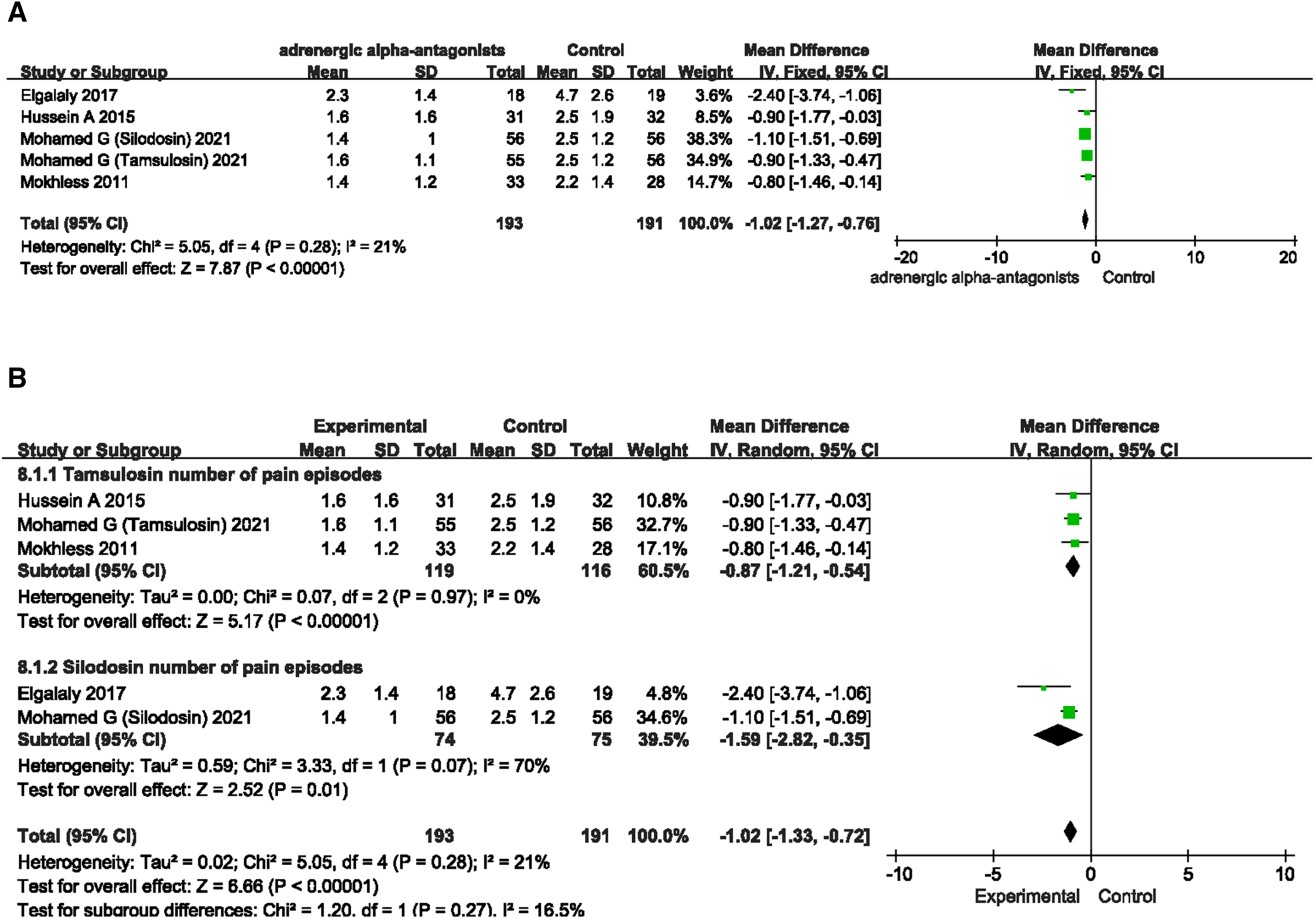
Forest plot comparing changes in number of pain episodes between adrenergic 443 alpha-antagonists and control groups. (**A**) Effects of treatment with adrenergic 444 alpha-antagonists on number of pain episodes compared with control group. (**B**) Subgroup 445 analysis of the effects of adrenergic alpha-antagonists on the number of pain episodes.

The 6 included RCTs and 1 cohort study all counted various TEAEs in adrenergic alpha-antagonists (including hypotension, asthenia, syncope, palpitations, somnolence, nausea, vomiting, headache, nasal congestion, and dizziness). We counted TEAEs in all included articles and analyzed the effects of the use of various adrenergic alpha-antagonists on the probability of TEAEs in children with ureteral stones. The results of the forest plot indicated that the probability of TEAEs during application of adrenergic alpha-antagonists was significantly increased compared with the control group, and the pooled estimate of the Risk ratio (RR) was 2.34, and the 95% confidence intervals (CI) was 1.40–3.91 (*p* = 0.001) (Figure 6A). We further divided the adrenergic alpha-antagonists group into three subgroups (tamsulosin group, doxazosin group and silodosin group) according to the types of drugs in the experimental group (Figure 6B). We found that the use of tamsulosin resulted in a significantly increased probability of TEAEs in children with ureteral stones (RR 2.26; 95% CI 1.21 to 4.22; *p* = 0.01). We analyzed these results and made a conclusion that ADRs should be considered when using tamsulosin. However, the application of doxazosin and silodosin had no statistically significant effect on the probability of TEAEs in children with ureteral calculi. This has a certain guiding effect for us on the selection of adrenergic alpha-antagonists in clinical practice.

## Discussion

Although there are differences between different countries and regions of the world, the overall incidence rate of ureteral calculi in children continues to rise every year. Relevant statistics show that the global incidence of urolithiasis in children is approximately 0.1%–5% (20). Genetic or systemic diseases (cystinuria or nephrocalcinosis), which account for approximately 17% in children, are relatively common identified etiologies (21). In addition, the heterogeneity of incidence rates in different regions suggests a significant influence of lifestyle and living habits. In principle, treatment strategies for pediatric patients are generally similar to those for adults, including observation, drug row stones, endoscopy, surgery and other methods (3). However, invasive treatment options such as endoscopy and surgery carry the additional risk of infection, submucosal tear, perforation, postoperative stenosis or obstruction, and other complications (22). Moreover, the risk of anesthesia and the cost of the procedure must be considered, especially in children. Meanwhile, pediatric patients may be at risk of low tolerance and poor compliance, which also brings difficulty in treatment (23). Therefore, a potential non-invasive treatment is usually considered the first choice for pediatric patients.

Medical expulsive therapy (MET) is one of the treatments for small-diameter stones in adults, especially <10 mm, which can avoid complications caused by non-drug interventions (24). The use of adrenergic alpha-antagonists can effectively promote stone expulsion and relieve pain, and is therefore considered a first-line agent in MET to promote exclusion of distal ureteral calculi (25, 26). The Urological Association of Asia clinical guideline for urinary stone disease (2019) states that adrenergic alpha −1 antagonists are an option for the treatment of distal ureteral stones >5 mm in diameter in adults (27). Although off-label use in children has been reported in several countries around the world, it has been shown in several studies to promote the transit of ureteral stones, especially distal ureteral stones <10 mm. Sridharan K and Ma L successively performed meta-analysis based on recent studies regarding efficacy and safety of different types of adrenergic alpha-antagonists in the treatment of ureteral calculi (28, 29). These trials confirmed, to varying degrees, the beneficial effects of adrenergic alpha-antagonists in the treatment of ureteral calculi. However, these studies focused mainly on adults and only a limited number of children were included. Therefore, the subgroup analysis of the two trials did not show a significant benefit of adrenergic alpha-antagonists in the treatment of ureteral calculi in children. An RCT study by Aydogdu O et al. on the efficacy of doxazosin in the treatment of ureteral calculi in children shows that hat daily treatment with doxazosin has no significant effect on the expulsion of distal ureteral calculi less than 10 mm in children (13). Another RCT study later confirmed that doxazosin could promote the expulsion of ureteral calculi, and relieve pain caused by ureteral calculi (15). In addition, this study also suggests that the therapeutic effect of doxazosin in children with ureteral calculi was different in different age groups. Furthermore, age played a role, as children under the age of 6 performed better than children over the age of 6. The study by Tuerxun A et al. has already shown that thinner ureteral walls and smaller stone sizes suggest a better treatment of ureteral stones in children with adrenergic alpha-antagonists (30). Younger pediatric patients tend to have thinner ureteral walls and smaller ureteral diameters, which may explain the better prognosis of younger pediatric patients treated with adrenergic alpha-antagonists. Several RCTs have confirmed that tamsulosin can effectively reduce the time of ureteral stone expulsion and increase the rate of ureteral stone expulsion in children (14, 16). Similarly, a multi-institutional cobot study by Tasian GE et al. also confirmed that tamsulosin improved the rate of ureteral stone expulsion in children, but the effect on stone expulsion time was not clarified (19). Elgalaly H et al. conducted an RCT study in 2017 to evaluate the efficacy of silodosin (a new type of adrenergic alpha-antagonist), and found that silodosin had good efficacy in treating ureteral calculi in children (20). Recently, Soliman MG et al. compared the treatment of silodosin and tamsulosin, and the results suggested that silodosin provides better stone expulsion effect than tamsulosin. At the same time, ADRs indicate the safety of these drugs in the treatment of ureteral calculi in children (17). There are differences between these trials in the placebo control, the type and dose of drugs, and the various biases considered, which may also lead to different results between the various trials. To investigate the efficacy and safety of adrenergic alpha-antagonists in the treatment of ureteral calculi in children, Tian, D. included several trials on tamsulosin and doxazosin in the treatment of ureteral calculi in children and conducted a meta-analysis, which suggested that the two drugs may improve the ureteral stone expulsive rate (10). However, due to the limited number of articles included, the effect of this study on stone expulsion time and drug-related ADRs were not clearly clarified. Therefore, given the heterogeneity of the results of all the above studies and the interest in the safety and efficacy of different drugs, we explore the effects of three different adrenergic alpha-antagonists on the time or rate of ureteral stone exclusion, number of pain episodes and TEAEs in children. We aim to further investigate the efficacy and safety of three different adrenergic alpha-antagonists in the treatment of ureteral calculi in children, in order to effectively self-discharge stones and avoid a series of complications caused by intervention or surgery.

Our meta-analysis showed that adrenergic alpha-antagonists were generally superior to placebo for the treatment and relief of symptoms of ureteral calculi in children. Tamsulosin, doxazosin and silodosin were effective in increasing the expulsive rate of ureteral calculi in children. Besides, tamsulosin and silodosin could also shorten the expulsive time of calculi and relieve the pain caused by calculi. Treatment with doxazosin and silodosin had no statistical significance on the incidence of ADRs, whereas tamsulosin significantly increased the incidence of ADRs in children with ureteral calculi. The above-mentioned results suggest that we should pay close attention to the ADRs caused by adrenergic alpha-antagonists during the treatment.

The limitation of this analysis was that some of the included trials lacked placebo control and were not blinded. Besides, although the stone size <10 mm was considered in our study, the stones larger than 10 mm were not discussed. If the size of stones over 10 mm could be included, the effect might be more obvious. Thirdly, adrenergic alpha-antagonists have dose effects (14). Therefore, it was worthwhile for us to use dose gradients in future studies to explore the relationship between adrenergic alpha-antagonists and stone self-dissolution. Fourthly, due to the limited number of trials including age stratification, we cannot obtain more pediatric patients to include in a meta-analysis to investigate the impact of child age on the treatment effect of adrenergic alpha-antagonists. Finally, compared with other treatment modalities, the optimal objective evaluation criteria for this treatment in the clinical course were less clear. Although further studies may be needed to explore the optimal dosage, the usage timing, and methods of treatment, our study proofed the efficacy and safety of three adrenergic alpha-antagonists in the treatment of ureteral calculi in children. We believed that this study could provide a valuable reference for the treatment of ureteral calculi in children with different types of adrenergic alpha-antagonists.

## Conclusions

In conclusion, adrenergic alpha-antagonists can not only promote the expulsion of ureteral calculi, but also reduce the number of pain episodes caused by ureteral calculi in children. However, the above mentioned effects differ to varying degrees between different types of adrenergic alpha-antagonists. Treatment with doxazosin and silodosin had no statistical significance on the incidence of ADRs, whereas tamsulosin significantly increased the incidence of ADRs in children with ureteral calculi. Therefore, ADRs caused by the use of three adrenergic antagonists remain a concern in clinical practice.

## Data Availability

The original contributions presented in the study are included in the article/Supplementary Material, further inquiries can be directed to the corresponding author/s.
